# The Thyroid–Metabolism Axis: Pathways of Dysregulation and the Effects of Treatment

**DOI:** 10.3390/metabo16040267

**Published:** 2026-04-16

**Authors:** Martina Curcio, Royce P. Vincent

**Affiliations:** 1 Faculty of Medicine and Surgery, Università Cattolica del Sacro Cuore, UCSC, 00168 Rome, Italy; martina.curcio02@icatt.it; 2 King’s College Hospital NHS Foundation Trust, London SE5 9RS, UK; 3 Faculty of Life Sciences and Medicine, King’s College, London SE1 7EH, UK

**Keywords:** thyroid dysfunction, metabolomics, lipidomics, cardiometabolic risk, subclinical thyroid disease, Graves’ disease, thyroid eye disease, treatment response, prognostic biomarkers

## Abstract

Thyroid hormones regulate a complex and interconnected network of metabolic signaling. Thyroid dysfunction is, at present, defined and monitored through circulating thyroid-stimulating hormone (TSH) and free thyroid hormones. However, biochemical normalization does not entirely indicate restoration of metabolic homeostasis. This discrepancy highlights a critical limitation of the current TSH-centric paradigm, which also fails to explain the heterogeneity in cardiometabolic outcomes observed among patients with similar biochemical profiles. Metabolomics, through the analysis of tissue-specific biofluids, could aid in capturing the complex metabolic perturbations that characterize this disease. In this review, we summarize metabolomic signatures typical of thyroid dysfunction, perform a critical evaluation of limitations and variability across studies, and explore the clinical and translational implications of metabolomics in thyroid pathology. In addition, five metabolic hubs influenced by thyroid hormone activity are summarized: (i) lipid and lipoprotein remodeling; (ii) mitochondrial energetics and redox balance; (iii) amino acid metabolism and protein turnover; (iv) gut–liver–thyroid axis and (v) biological impact of subclinical thyroid diseases. Taken together, these findings challenge the sufficiency of a diagnostic model based on TSH measurement and pose metabolomics as a promising tool to refine risk stratification, uncover subclinical vulnerability and guide patient-centered management of thyroid disease. Despite its promise, clinical adoption of metabolomics is hindered by a lack of standardization and complex data interpretation. To overcome these limitations, coupling metabolomics with genomics and transcriptomics may allow its translation into practical application.

## 1. Introduction

Human metabolism is deeply influenced by thyroid hormones, which regulate protein turnover, thermogenesis, and lipid and glucose handling. Therefore, the systemic effects on the liver, skeletal muscle, adipose tissue and bone resulting from thyroid diseases extend beyond endocrine balance and determine changes in body composition, leading to enhanced cardiovascular risk and tissue-specific pathology [1].

**Figure 1:**
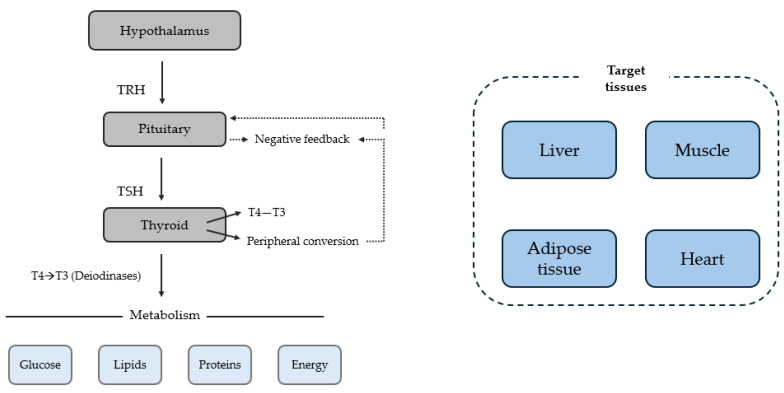
The Hypothalamic-Pituitary (HPT) Axis and its Systemic Metabolic Effects. Abbreviations: TRH, thyrotropin-releasing hormone; TSH, thyroid stimulating hormone; T4, thyroxine; T3, triiodothyronine.

The activity of thyroid hormones is strongly dependent on the hypothalamic–pituitary–thyroid (HPT) axis (Figure 1). The first regulator of this system is thyrotropin-releasing hormone (TRH), which is secreted by the hypothalamus. It stimulates the anterior pituitary gland (located at the base of the brain directly beneath the hypothalamus) to produce thyroid-stimulating hormone (TSH), which in turn promotes the synthesis and release of thyroxine (T4) and triiodothyronine (T3) from the thyroid gland.

Circulating T4 and T3 exert negative feedback on both the hypothalamus and the pituitary, limiting their own synthesis and maintaining hormonal homeostasis. The activity of T3 is further regulated by deiodinases, which determine the tissue-specific availability of T3 [1,2].

Any disruption of the HPT axis may result in thyroid dysfunction, both subclinical and overt, forming the biochemical basis for the measurement of TSH and free thyroid hormones in clinical practice.

Management of endocrine diseases is heavily dependent upon laboratory evaluations and available technologies. Accordingly, current clinical assessment of thyroid pathology relies prevalently on circulating concentrations of TSH and free thyroid hormones (FT3 and FT4). Furthermore, thyroid autoantibodies such as thyroid peroxidase antibodies (TPO) and thyroid-stimulating hormone receptor antibodies (TRAb) are measured to guide the differential diagnosis of autoimmune thyroid disease [3]. The accuracy of such measurements is crucial for definitive diagnosis and treatment of thyroid disorders. Nonetheless, these capture neither the complexity of the downstream metabolic pathways involved nor the inter-individual variability in clinical outcomes.

Metabolomics is a rapidly evolving diagnostic laboratory technology that aims to detect small molecules in different sample matrices such as blood, urine and tissue [4]. It can comprehensively identify endogenous and exogenous low-molecular-weight (<1 kDa) molecules or metabolites in a high-throughput manner [5]. The composition of endogenous compounds is affected by both proteome and genome, as well as lifestyle factors, environment, medication and underlying disease [4,5].

Therefore, by interrogating amino acids, lipids, energy and immune-related pathways simultaneously using nuclear magnetic resonance spectroscopy (NMR) or mass-spectrometry-based (MS) platforms, metabolomics could aid in understanding the sources of biological variability in thyroid dysfunction and treatment effects [4,5].

This tool has been increasingly applied to endocrine disorders and has demonstrated its potential in both identifying disease-specific metabolic features and improving phenotypic stratification. Metabolomic profiling and its recent applications in endocrinology allow the characterization of complex metabolic networks that are not captured by standard hormonal assays, making it relevant for future, patient-centered clinical practice [6].

In the context of thyroid disease, metabolomics could enable the detection of subtle metabolic alterations that may not be reflected by conventional biochemical markers. This becomes particularly relevant in light of the recognized discrepancy between biochemical euthyroidism and persistent metabolic dysregulation. Consistent with this, Jaber et al. (2022) showed that metabolomic analyses in subjects treated for hyperthyroidism reveal persistent alterations in lipid and energy metabolism despite biochemical euthyroidism, highlighting the residual metabolic disturbances that may remain undetected by standard thyroid function tests [7].

Beyond overt conditions, the high sensitivity of metabolomics may be useful in a more precise characterization of subclinical thyroid dysfunction, which is becoming increasingly clinically and therapeutically relevant. Accordingly, Shao et al. demonstrated that patients with subclinical hypothyroidism present relevant alterations in both lipid and energetic pathways, despite normal thyroid hormone concentrations, suggesting that metabolic abnormalities may precede overt biochemical alterations [8].

In this context, by incorporating metabolomic insights, our review aims to integrate current evidence on the thyroid–metabolism axis. Specifically, we aim to (i) summarize metabolomic signatures typical of thyroid dysfunction, (ii) perform a critical evaluation of limitations and variability across studies, and (iii) explore the clinical and translational implications of metabolomics in thyroid pathology. In addition, five metabolic hubs influenced by thyroid hormones’ activity are summarized: (i) lipid and lipoprotein remodeling; (ii) mitochondrial energetics and redox balance; (iii) amino acid metabolism and protein turnover; (iv) gut–liver–thyroid axis and (v) biological impact of subclinical thyroid diseases.

## 2. Results

### 2.1. Thyroid Dysfunction: Clinical Background

Thyroid dysfunction could be considered a spectrum, involving hyperthyroidism, biochemically defined as an excess of circulating hormones in the blood, and its opposite, hypothyroidism, as well as subclinical states defined by abnormal TSH and within-range circulating free thyroid hormones. Subclinical forms are particularly prevalent in older populations [3], whereas overt disease, though less represented, carries substantial morbidity [3].

Although both hyperthyroidism and hypothyroidism are common worldwide, reported prevalence ranges from 0.2 to 1.3% for overt hyperthyroidism and 0.3 to 0.8% for overt hypothyroidism, with a notable geographic variation [3], reflecting differences in iodine availability across the various areas of the world, a trace element from which thyroid hormones are synthesized.

The daily iodine intake in adults varies from less than 10 µg in areas of extreme deficiency to several hundred milligrams for those receiving medicinal iodine. Whilst dietary iodine is a key determinant of thyroid disease risk, other factors, such as aging, genetic susceptibility, ethnicity, smoking status, endocrine disruptors and the advent of novel therapeutics such as immune checkpoint inhibitors, also influence thyroid disease epidemiology [3]. With iodine imbalance, toxic nodular thyroid disease and iodine-deficient hypothyroidism are more frequent, whereas the most represented cause overall consists of autoimmune forms of the disease.

Women are disproportionately affected by thyroid conditions, reflecting the autoimmune background of many cases [3]. This is also relevant in the management of thyroid status in women of childbearing age, as pregnancy has profound effects on the regulation of thyroid function in healthy women and patients with thyroid disorders. Overt thyroid dysfunction occurs in 2–3% of pregnancies, but subclinical thyroid dysfunction (both hyper- and hypothyroidism) is probably more prevalent and frequently remains undiagnosed, unless specific screening programs are initiated to disclose thyroid function abnormalities in early gestation [9,10]. Pregnancy may affect the course of thyroid disorders (due to altered iodine status or thyroid antibodies) and, conversely, thyroid diseases may affect the course of pregnancy. Consequently, they may affect both the pregnant woman and the developing fetus [9,10]. In addition, the reliance on TSH concentrations, especially if not interpreted using trimester-specific reference limits, can have an adverse impact on mother and fetus [11].

Congenital hypothyroidism (CH) is defined as a variable dysfunction of the hypothalamic–pituitary–thyroid axis present at birth. CH can have major detrimental effects on growth and neurological development, but early intervention leads to excellent outcomes [12]. Therefore, neonatal screening is essential for the early diagnosis of CH. A blood sample is collected from the newborn’s heel on a Guthrie card that is sent to the laboratory for analysis. Most screening programs measure TSH concentrations with high sensitivity, but up to 10% return false-negative results [12]; hence, metabolomics could play a key role in CH screening with adequate research and implementation.

### 2.2. Metabolomic Signatures in Thyroid Diseases: Analytical Approaches and Clinical Insights

In thyroid diseases, metabolomic investigations are conducted using NMR spectroscopy and MS-based platforms. The basic principles of NMR are that the structural and chemical composition of different substances can be determined by their nuclei, which have their distinctive magnetic fields [13]. This technique offers many advantages such as non-invasiveness (the studies of biological cells and tissues are now possible without damaging the sample), avoids exposure to radiation, which could be harmful to both the researcher and the patients (this can ensure safety and reduce experimental costs due to the removal of discarded radioactive substances), flexibility for wide application (extensive processes can be investigated to acquire a wide variety of information) and detailed structural analysis. On the other hand, it is a very costly technique and requires a high magnetic field to perform the analysis [13].

NMR has proven to be highly suitable for large-scale studies due to its high reproducibility and minimal sample preparation requirements; however, it is limited by lower sensitivity. In contrast, broader metabolite coverage and higher sensitivity are typical of MS-based methods, especially if coupled with liquid chromatography (LC-MS), although greater analytical variability characterizes them.

Metabolomics captures downstream functional alterations that reflect the combined effects of modified genomics, transcriptomics and proteomics, representing indeed the most integrative level of biological regulation [5,14]. Within this framework, both untargeted and targeted metabolomic approaches have been applied in the field of thyroid research. Untargeted metabolomics offers the identification of global metabolic perturbations, while targeted approaches focus on the quantitative measurement of predefined metabolites, thereby facilitating clinical interpretation.

Jaber et al. (2022) showed that untargeted metabolomics is particularly useful for the identification of lipid remodeling and amino acid metabolism, whereas targeted approaches may aid in the validation of specific metabolites with potential clinical relevance [7]. In particular, phosphatidylcholines, sphingolipids, and lipoprotein subclasses have been implicated in the context of enhanced cardiometabolic risk, due to their well-known involvement in membrane structure and inflammatory signaling pathways. Phosphatidylcholines may affect both functionality and composition of lipoproteins, being associated with impaired lipid transport and the promotion of atherogenic profiles. A similar role is played by sphingolipids, including ceramide-related species, that promote endothelial dysfunction, oxidative stress and, therefore, a pro-inflammatory state [7,15]. Collectively, these observations provide the basis for interpreting the metabolic changes associated with different thyroid functional states.

Overt hyperthyroidism is best identified through its hypermetabolic features, such as increased metabolic rate, augmented lipolysis and accelerated protein catabolism. This is highlighted by studies focusing on its metabolic profile. Research consistently shows shifts in lipoprotein subclass composition, as well as lipid remodeling, including altered phosphatidylcholines and sphingolipids [7] (Table 1).

An augmented catabolic activity is also represented by increased proteolysis, with consequent reduction in branched-chain amino acids (BCAAs). Contemporarily, the presence of energy-related metabolites indicates an increased oxidative stress and mitochondrial activity [7]. Part of hyperthyroidism’s significant morbidity is determined by typical cardiovascular manifestations including atrial fibrillation and heart failure, making it a potentially life-threatening presentation [15,16].

An opposite metabolic pattern is present in hypothyroidism, which is characterized by reduced energy expenditure, weight gain, and dyslipidemia. Beyond elevations in conventional lipid markers, such as low-density lipoprotein (LDL) and triglycerides, lipid profiling reveals atherosclerotic remodeling of lipid species and lipoprotein particles, consistent with increased cardiovascular risk (Table 1). Heterogeneous alterations in mitochondrial energetics and amino acid metabolism are also seen, suggesting impaired metabolic efficiency.

In subclinical conditions, subtle but reproducible lipidomic and energetic alterations overlap with overt disease signatures (Table 1), challenging the assumption that this kind of dysfunction is metabolically mild [8]. Subclinical thyroid disease is defined as such in the presence of altered TSH and circulating thyroid hormone parameters within the reference range. Both subclinical hyperthyroidism and subclinical hypothyroidism exist, with the latter being the most common, with an overall prevalence of 3–10%. The subjects that are most affected by this condition are older women, whereas subclinical hyperthyroidism prevalence varies broadly across populations and iodine availability [3]. Even if free thyroid hormones (FT3 and FT4) remain within the normality range [17], abnormal TSH concentrations reflect an altered hypothalamus–hypophyseal–thyroid axis activity. This helps to understand why biochemically “mild” conditions can still significantly affect the systemic functioning of the human body.

The most common clinical implication of such dysfunction lies in cardiometabolic profile risk: TSH concentrations above 10 mIU/L have been associated with a higher risk of coronary heart disease and heart failure in younger subjects [18]. Moreover, a higher atherogenic profile is seen in the presence of a subclinical hypothyroidism state, due to higher concentrations of LDL and total cholesterol, increased diastolic dysfunction and enhanced peripheral vascular resistance.

By contrast, rather than atherogenic complications, subclinical hyperthyroidism is linked to arrhythmogenic and hemodynamic complications, especially if TSH concentrations are suppressed below 0.1 mIU/L [19]. Yet, subclinical states of the disease have only been explored in depth recently, and further research is required to delineate a more specific picture of their metabolic implications.

Importantly, the idea of dissociation between biochemical evidence and underlying metabolic dysregulation is not only limited to subclinical states but also applicable in overt thyroid diseases. In fact, although biochemical parameters are generally normalized through treatment, both in hyperthyroidism and hypothyroidism, some molecular abnormalities might persist despite TSH normalization, particularly involving lipid- and energy-related networks, highlighting potential residual metabolic risk after hormonal correction [7,20].

### 2.3. Integrative Overview of Metabolomic Alterations Across Thyroid States

At present, metabolomic evidence reveals that thyroid dysfunction, rather than representing a collection of isolated biochemical changes, implies a systemic reorganization of metabolic homeostasis. Thus, the body’s internal balance is affected as a whole by these coordinated, cross-pathway perturbations.

A pivotal observation in recent research is the decoupling of biochemical thyroid status from the underlying metabolic phenotype. In fact, persistent metabolic patterns remain detectable regardless of the normalization of hormonal levels. These metabolic fingerprints pertain to the entire clinical spectrum, including subclinical forms of the disease, suggesting that metabolic disruption may serve as a leading indicator, preceding or outlasting traditional endocrine markers.

Moving beyond independent chemical fluctuations, these changes converge upon key metabolic hubs both at a systemic and tissue-specific level, possibly explaining the heterogeneous clinical manifestation observed across the different body systems. They involve lipid and lipoprotein profiles, mitochondrial energetics and redox stability. Furthermore, a determinant role is played by remodeling of amino acid metabolism and gut–liver signaling axis, while the tissue-specific modifications take place mainly in the skeletal and orbital compartments.

#### 2.3.1. Lipid and Lipoprotein Remodeling

Altered lipid and lipoprotein metabolism are one of the most consistent signatures across hyperthyroidism, hypothyroidism and subclinical conditions [7,8,20]. Recent studies have demonstrated qualitative alterations in sphingolipids, phospholipids and lipoprotein particle compositions that reflect changes in membrane dynamics, lipid trafficking and activation of inflammatory pathways. Consequently, lipid dysfunction can be considered a condition going beyond quantitative changes in total cholesterol and triglycerides [7,8,20]. This might help to understand the well-established association between thyroid dysfunction and cardiovascular diseases [15,16], and to explain why normalization of thyroid hormone concentration does not invariably translate into lowering of cardiovascular risk.

Other studies support the idea of residual metabolic risk despite biochemical euthyroidism, as denoted by evidence of persistent dyslipidemia in post-therapeutic profiling of some hyper- and hypothyroid patients [7,20]. This highlights how metabolomics might be fundamental in revealing subclinical atherogenic remodeling that is still not fully addressed by traditional lipid panels.

#### 2.3.2. Mitochondrial Energetics and Redox Balance

Thyroid hormones actively regulate mitochondrial biogenesis and oxidative phosphorylation. Increased mitochondrial flux and oxidative stress are particularly relevant in hyperthyroidism, which is characterized by alterations in glycosidic intermediates and tricarboxylic acid (TCA) cycle-related metabolites. Instead, hypothyroidism is associated with a distinct metabolic panel, consistent with impaired mitochondrial function, reduced energetic activity and altered redox balance [7,8,20].

Current research supports the idea that the correction of hormonal levels does not necessarily translate into remission, and both the ongoing symptoms and cardiometabolic vulnerability in a subset of patients can be explained by this constant dysregulation of energy-related metabolites [7,20]. Accordingly, inevitable implications for treatment monitoring are present, supporting the need for broader metabolic endpoints in thyroid disease management.

#### 2.3.3. Amino Acid Metabolism and Protein Turnover

Hyperthyroidism is associated with alterations in amino acid and protein metabolism. Reduced concentrations of BCAAs can be seen in this disease, underscoring an increased energy demand and proteolysis. Conversely, more heterogeneous perturbations are observed in hypothyroidism, reflecting the variable effects on insulin sensitivity, nitrogen balance and muscle metabolism [7,8,20]. These findings underscore inter-individual metabolic heterogeneity and might help to delineate the divergent clinical trajectories among similar thyroid hormonal profiles, suggesting potential relevance for risk stratification.

#### 2.3.4. Gut–Liver–Thyroid Axis

An emerging yet under-explored component of the thyroid–metabolism axis is represented by bile acid metabolism. Bile acid pathways regulating glucose and lipid metabolism, such as those involving farnesoid X receptor (FXR) and Takeda G protein-coupled receptor 5 (TGR5), are influenced by circulating thyroid hormones. Additionally, the bidirectionality of this interaction is highlighted by altered concentrations of conjugated bilirubin in thyroid dysfunction [7]. Furthermore, gut microbiota plays an important role in systemic immune homeostasis and is increasingly implicated in autoimmune thyroid disease [21]. Some studies have suggested that gut dysbiosis, impaired intestinal barrier function, and altered microbial metabolites, particularly short-chain fatty acids, contribute to immune imbalance along the gut–thyroid axis [21,22]. However, current evidence remains limited, and a biologically plausible link cannot be excluded.

#### 2.3.5. Biological Impact of Subclinical Thyroid Diseases

Despite its recognized association with arrhythmia and cardiovascular risk [19] in the overt thyroid disease, metabolomic characterization of subclinical hypothyroidism remains scarce. Recent studies are revising the assumption that subclinical states are metabolically silent. In fact, subtle yet reproducible lipid and energetic alterations are appreciated in subclinical hypothyroidism, overlapping, to a certain extent, with those of the overt disease. These findings might support the hypothesis that metabolic dysregulation may precede overt hormonal abnormalities [8]. By studying molecular profiles in this population, it could be possible to better identify biologically relevant subgroups and refine treatment strategies, going beyond TSH-centric decision-making. However, from a clinical perspective, metabolomics is not yet implemented in standard diagnosis and monitoring. Major barriers limiting its use have been pointed out by recent translational studies and include, beyond the lack of standardized protocols, high costs and technical complexity [4,23].

Nevertheless, certain organ-specific dysfunctions resulting from the detrimental effects of altered thyroid hormones could benefit from an early and precise detection offered by metabolomics. This is particularly valid for skeletal disease and Thyroid Eye Disease (TED), mainly because, in such cases, prompt detection and management may change disease progression and severity in the long term, as well as decrease the overall healthcare burden associated with the advanced stages of these diseases.

### 2.4. Bone Metabolism and Skeletal Consequences of Thyroid Dysfunction

One of the most sensitive targets of thyroid hormone actions is bone. T3 acts mainly through thyroid hormone receptor alpha 1 (TRα1) expressed in skeletal tissue to mediate osteoblast differentiation and coupling between bone formation and resorption [24].

Accelerated bone turnover is common in hyperthyroidism and results from excessive thyroid hormone signaling, which shortens the remodeling cycle. Progressive reduction in bone mineral density (BMD), disruption of trabecular architecture and increased fracture risk due to increased osteoclastic activity contribute to bone fragility. Interestingly, similar findings have been seen in subclinical hyperthyroidism, suggesting that suppressed TSH states can still have relevant effects even in the absence of overt thyroid hormonal excess [25].

In contrast, prolonged remodeling cycles and delayed matrix mineralization have been reported in hypothyroidism. Although acute alterations in BMD may be absent, impaired bone quality and altered mechanical properties have been highlighted [25]. Notably, TSH could have a direct inhibitory effect on osteoclastogenesis, regardless of circulating thyroid hormone concentrations. Therefore, in suppressed TSH states, skeletal effects may involve both hormone-dependent and hormone-independent pathways [24,25].

Apart from standard diagnostic tools for BMD assessment, such as Dual-Energy X-ray Absorptiometry (DXA) scan, serum markers such as C-terminal telopeptide of type 1 collagen (CTX), reflecting bone resorption, and procollagen type 1 N-terminal pro-peptide (P1NP), reflecting bone formation, are now increasingly used. Integration of these biomarkers within broader metabolomic profiling might not only give important information about tissue-level recovery following restoration of biochemical euthyroidism but also identify residual skeletal risk.

### 2.5. Thyroid Eye Disease

TED is an autoimmune disorder leading to functional and visual impairment. Determinant factors in its pathogenesis include inflammation, increased oxidative stress, adipogenesis and fibrosis, resulting in eye symptoms such as foreign body sensation and decreased visual acuity [26]. Distinct metabolites can be seen in serum and tear fluid of the affected patients (Table 2) with different concentrations according to the disease activity, creating a biological background for new potential clinical scoring systems [27,28,29].

TED management has been deeply changed by the advent of molecular agents such as teprotumumab (Tepezza^®^), an insulin-like growth factor 1 (IGF-1) receptor inhibitor. The use of this monoclonal antibody led to an expansion of treatment indications, which now include chronic and low-activity disease [30], underscoring the need for biomarkers capable of predicting not only treatment response but also durability and relapse risk.

Together with IGF-1, tryptophan-derived immunomodulatory pathways are emerging as possible contributors to oxidative stress and aberrant amino acid metabolism, eventually associated with immune activation and orbital fibroblast dysfunction typical of TED. As extensively discussed in reviews of Graves’ ophthalmopathy, IGF-1 and tryptophan-related pathways represent promising candidates for future prognostic research and stratification [31,32].

### 2.6. Metabolomics to Multi-Omics

To achieve the full clinical applicability of metabolomics, it becomes necessary to integrate it with complementary tools, such as genomics, transcriptomics and proteomics information. In this way, metabolic alterations could be properly contextualized within their underlying regulating and upstream networks. Consequently, both the interpretability and clinical relevance of metabolomics may be increased [14,23,33].

In particular, in thyroid pathology, where the mismatch between circulating hormones and clinical and/or metabolic outcomes is frequent, this multi-omics model may improve patient stratification, so that, despite similar TSH concentrations, subgroups with different cardiometabolic risk profiles could be identified [8,33].

Consequently, it may become possible to identify high-risk subclinical conditions and to predict tissue-specific complications, which include skeletal involvement and thyroid eye disease. Additionally, within this multi-omics framework, it might be useful to integrate microbiome-derived signals to refine the understanding of inter-individual variability in disease expression [21,22,33].

## 3. Discussion

The present review highlights how metabolomics enables more rapid discovery and validation of metabolic indicators of disease. Metabolomics has been shown to be a powerful approach to capture the integrated downstream consequences of altered thyroid signaling, through which it is possible to underpin both cardiometabolic risk and tissue-specific manifestations [4,7,8].

**Figure 2:**
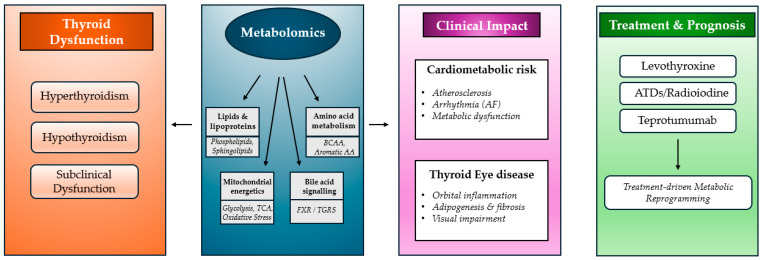
Metabolomics as a translational interface between thyroid dysfunction and clinical phenotypes. Abbreviations: BCAA, branched-chain amino acids AA, amino acids; TCA, tricarboxylic acid; FXR, farnesoid X Receptor; TGR5, Takeda G-protein–coupled Receptor 5; AF, arterial fibrillation; ATDs, anti-thyroid drugs.

The metabolic reorganization observed in thyroid dysfunction converges on specific “hubs” that explain the heterogeneity of the clinical spectrum. Coordinated perturbations across lipid metabolism, mitochondrial energetics, amino acid turnover, bile acid signaling and immune-metabolic pathways can result from the effects of thyroid hormones on multiple metabolic nodes and determine the detrimental effects of this system-level metabolic condition (Figure 2).

Our analysis of lipid and lipoprotein remodeling suggests that thyroid-driven dyslipidemia transcends quantitative shifts in cholesterol, thus involving qualitative alterations in phospholipids and sphingolipids, and promoting a pro-atherogenic and inflammatory environment. This provides a biological rationale for the persistent cardiovascular risk observed even in biochemically normalized subjects. Similarly, a metabolic substrate for both the hypermetabolic state of hyperthyroidism and the hypometabolic state observed in hypothyroidism may be offered by altered mitochondrial energetics and redox balance. This potentially explains the persistence of symptoms among some patients, despite within-range TSH concentrations. Furthermore, inter-individual variability in muscle mass and nitrogen balance seems to be linked to systemic catabolic and anabolic shifts driven by changes in amino acid metabolism and protein turnover. A particularly novel insight pertains to the role of the gut–liver–thyroid axis, where bile acid-mediated signaling (via FXR and TGR5) may act as a bridge between endocrine status and systemic health, likely influenced by host microbiome.

According to our findings, thyroid dysfunction leaves unique signatures in the skeletal and orbital systems: in bone, metabolic profiling could identify residual fracture risk, which may be missed by standard DXA scans, by capturing the accelerated or delayed turnover cycles directly at a biochemical level; additionally, the identification of specific metabolites in tears and serum, such as those related to oxidative stress and tryptophan metabolism, represent a promising avenue for both disease and therapeutic monitoring in TED.

Crucially, our review challenges the belief that subclinical states are metabolically silent. Present research suggests that, in subclinical patients, subtle yet reproducible metabolic alterations are found, and this metabolic disruption may even precede overt biochemical failure. The presence of this “metabolic memory” underscores the insufficiency of the current TSH-centric model for comprehensive patient risk stratification.

Despite the growing interest in metabolomics and its application to understand the cellular metabolic effects in endocrine diseases such as thyroid pathology, several challenges characterize this approach.

Specifically, for the study of thyroid diseases, the current application of metabolomics remains limited at the level of available evidence. Using metabolomic profiling, Shao et al. [8] demonstrated that patients with both subclinical and overt hypothyroidism present with similar serum metabolic profiles. This data questions the current American Thyroid Association and European Thyroid Association guidelines [34,35], which advise treating subclinical hypothyroidism with Levothyroxine if TSH > 10 mIU/L and/or relying upon individual patient symptoms. Although symptoms in subclinical hypothyroidism do not always improve with Levothyroxine treatment, long-term metabolic outcomes may respond in certain patients after treatment [20,36]. Furthermore, Piras et al. [20] showed that patients with newly diagnosed hypothyroidism restored serum TSH and FT4 concentrations within three months of treatment, but their baseline metabolomic profiles remained essentially unchanged. These results emphasize the shortcomings of relying solely on current biomarkers to monitor the efficacy of Levothyroxine treatment to correct thyroid status, since individual tissues may lag temporally behind the HPT axis in returning to euthyroid status. Thus, further clinical studies utilizing metabolomics may help to improve long-term clinical management in this cohort of patients.

Most studies focus only on specific metabolic pathways, primarily lipid metabolism, amino acid turnover or mitochondrial energetics, missing an integrated system-level perspective. In addition, as highlighted by Clish et al. (2015), the limited number of studies available use different analytical platforms (NMR versus LC-MS), sample types, and study designs, elements that contribute to variability in reported findings and limited comparability across studies [4]. Reproducibility is thereby another key issue, since variability in both sample collection and processing deeply influences metabolomic outputs [4]. Furthermore, the studies evaluating these techniques have utilized relatively small cohorts and cross-sectional designs, and this represents a relevant barrier to biomarker validation [4,23]. Collectively, these challenges have currently limited the application of metabolomics to research studies. However, our review highlights its potential to advance personalized medicine if utilized appropriately in diagnostic pathways, treatment monitoring and prognostication.

Nicholson et al. shed light on other confounders [22]. A complex element that needs to be considered is represented by human microbiomes. Metabolite levels are influenced by both internal and external factors, such as lifestyle and infections: host–microbiome interactions could thereby alter disease-related metabolomic features, pharmacotherapy and malignancy. Moreover, comorbidities such as obesity and cardiovascular diseases themselves might alter metabolomic profiles, making it difficult to isolate the specific metabolomic contributions of thyroid pathology.

Therefore, the full potential of metabolomics in thyroid diseases is still not completely realized. The development of defined clinical guidelines, precise methodological approaches and standardized analytical frameworks guiding its use will be fundamental in establishing it as a reliable and powerful tool to uncover residual disease signatures and subclinical conditions. Additional requirements for wider application will be influenced by cost-effective and routinely accessible metabolomic applications to replace the conventional biomarkers currently in use.

Nevertheless, through heterogeneous studies, converging evidence supports the presence of recurrent metabolic signatures, enabling the identification of biologically relevant pathways typical of thyroid dysfunction [7,8,20].

Taken together, these findings advocate for a paradigm shift: moving away from a strictly hormone-centric model toward a systems-level interpretation of thyroid diseases, accounting for their broader metabolic complexity.

### Limitations

This review itself has some limitations. The literature analyzed is heterogeneous in terms of reported analytical platforms, sample types and study designs, resulting in partly exploratory conclusions. Further validation through larger and better characterized cohorts is thus needed to establish clinical utility and the full potential of metabolomics in the routine care of thyroid disease management.

Our review does not include the applications and advances made in metabolomics utilized for risk stratification and identification of therapeutic targets in the management of thyroid lesions and thyroid malignancies [37,38,39]. Our narrative is focused on metabolomics in benign thyroid diseases.

## 4. Conclusions

Our evolving understanding of the complex metabolic interplay of thyroid dysfunction highlights the need to consider new technological tools such as metabolomics as key determinants for thyroid disease characterization. Across overt and subclinical conditions, the evidence reviewed here indicates that both the diagnosis and prognosis of thyroid morbidity should expand beyond the standard serum biomarkers that are currently in routine use, with the goal of optimizing clinical management and outcomes.

Metabolomics provides an integrative output complementing conventional endocrine assessment and offers a steer toward more personalized and precise risk stratification and treatment monitoring. At the same time, the translation of the metabolomics approach into clinical practice remains currently limited by important challenges, both from a methodological and interpretative point of view. These include the lack of standardization, analytical variability, high cost, the use of small cohorts in the studies currently available, and the influence of confounding factors, such as upstream biological alterations. However, the integration of metabolomics with genomics, transcriptomics and microbiome-derived elements may provide a more robust biological context aimed at improving metabolic signatures interpretation, patient stratification and guiding new therapeutic strategies.

Future research should focus on standardized analytical platforms and methodology, prospective validation of biomarkers, and integration of multi-omics strategies to support the translation of metabolomics into a more patient-centered approach to medicine.

## Figures and Tables

**Table 1:** Metabolomic signatures across thyroid dysfunction phenotypes and treatment status.

Thyroid Status	Biospecimen	Analytical Platform	Main Altered Pathways	Representative, Metabolomic Features	Clinical/Translational Implications
Overt Hyperthyroidism	Serum/Plasma	NMR, LC–MS	Lipids & Lipoproteins	Remodeling of phosphatidylcholines and sphingolipids; altered lipoprotein subclass composition	Cardiometabolic risk not fully captured by standard lipid panel
			Mitochondrial Energetics	Increased glycolytic intermediates; altered TCA-cycle–related metabolites; oxidative stress signals	Hypermetabolic, pro-oxidative phenotype
			Amino Acids	Reduced branched-chain amino acids; altered aromatic amino acids	Enhanced proteolysis and nitrogen flux
			Bile Acids	Shifts in conjugated bile acids	Potential modulation of glucose–lipid signaling via FXR/TGR5
			Post-Treatment State	Partial normalization of lipid and energy pathways	Evidence of persistent metabolic remodeling (“metabolic memory”)
Overt Hypothyroidism	Serum/Plasma	NMR, LC–MS	Lipids & Lipoproteins	Increased LDL-related lipid species; altered phospholipid profiles	Atherogenic lipid remodeling
			Mitochondrial Energetics	Reduced energy flux; altered lactate/pyruvate ratios	Impaired mitochondrial efficiency
			Amino Acids	Variable perturbations across studies	Inter-individual metabolic heterogeneity
			Levothyroxine Response	Improvement of conventional lipid markers; persistence of some lipidomic features	Biochemical euthyroidism may not equal full metabolic normalization
Subclinical Hypothyroidism	Serum	NMR	Lipids & Energetics	Mild but consistent lipidomic shifts; subtle energetic alterations	Early cardiometabolic signal despite “subclinical” status
Subclinical Hyperthyroidism	Serum	Limited data	Energetics/ CV-related pathways	Incompletely characterized	Key unmet research area

Key metabolomic pathways altered in overt and subclinical thyroid dysfunction, with emphasis on lipid metabolism, mitochondrial energetics, amino acid turnover, bile acid signaling, and treatment-related metabolic reprogramming. Abbreviations: NMR, Nuclear Magnetic Resonance; LC–MS, Liquid Chromatography–Mass Spectrometry; TCA cycle, Tricarboxylic Acid cycle; LDL, Low-Density Lipoprotein; FXR, Farnesoid X Receptor; TGR5, Takeda G-protein–coupled Receptor 5; CV, Cardiovascular.

**Table 2:** Metabolomic and multi-omics insights in thyroid eye disease (TED): treatment and prognostic perspectives.

Clinical Aspect	Biospecimen	Omics Approach	Key Altered Pathways	Translational/ Prognostic Relevance
Disease Activity (Active vs. Inactive TED)	Tear fluid	Proteomics/ Metabolomics	Oxidative stress; immune–metabolic pathways	Differentiation of disease activity states
Systemic–Local Interaction Tryptophan Metabolism	Serum/Tears	Multi-omics	Inflammation–metabolism crosstalk	Links thyroid autoimmunity with orbital pathology
	Serum/ Experimental models	Metabolomics	Kynurenine and indole derivatives	Modulation of orbital fibroblast inflammation and proliferation
Oxidative Stress Biomarkers Treatment Response (Teprotumumab)	Serum/Tears	Targeted assays	Redox imbalance	Potential indicators of severity and progression
	Serum (emerging data)	Exploratory omics	IGF-1R–related metabolic signaling	Need for predictive biomarkers of response
Prognostic Gap	—	—	—	Identification of relapse risk and durability of response remains unmet

Summary of metabolomic and multi-omics findings in thyroid eye disease, with relevance to disease activity, treatment response, and prognostic biomarker development. Abbreviations: TED, Thyroid Eye Disease; IGF-1R, Insulin-like Growth Factor 1 Receptor; Multi-omics, integrated multi-layer molecular profiling (e.g., genomics, proteomics, metabolomics); Redox, reduction–oxidation balance; Kynurenine pathway, tryptophan degradation pathway involved in immune and inflammatory regulation.

## Data Availability

No new data were created or analyzed in this study.
